# Alcohol Trajectories over Three Years in a Swedish Residence Hall Student Population

**DOI:** 10.3390/ijerph7041432

**Published:** 2010-03-29

**Authors:** Henriettae Ståhlbrandt, Anders Leifman, Kent O. Johnsson, Mats Berglund

**Affiliations:** 1 Clinical Alcohol Research, Entrance 108, UMAS, SE-205 02 MALMÖ, Sweden; E-Mails: Kent.Johnsson@med.lu.se (K.O.J.); Mats.Berglund@med.lu.se (M.B.); 2 Karolinska Institute, Department of Public Health Sciences and Centre for Alcohol and Drug Prevention, Novum, SE-141 57 HUDDINGE, Sweden; E-Mail: anders.leifman@sll.se

**Keywords:** alcohol, college students, residence halls, long-term follow-up, social climate

## Abstract

Although it is known that college students have a high alcohol consumption, less is known about the long-term drinking trajectories amongst college students and, in particular, students living in residence halls, known to be high-risk drinkers. Over four consecutive years, the drinking habits of 556 Swedish residence hall students were analyzed. The main instruments for measuring outcome were AUDIT (Alcohol Use Identification Disorders Test), SIP (Short Index of Problems) and eBAC (estimated Blood Alcohol Concentration). The drinking trajectories among Swedish residence hall students showed stable and decreasing drinking patterns, with age and gender being predictors of group membership.

## 1. Introduction

### 1.1. College Student Alcohol Consumption

College students have high mean alcohol consumption and risky alcohol habits. American data show that approximately 42% of college students have one or more sessions of heavy episodic drinking in the past month [1]. The corresponding figure in Swedish college students is 55% [2]. Studies have shown that alcohol use varies according to accommodation arrangements, with high alcohol consumption reported in American fraternities and sororities [3–5], as well as in residence halls in Sweden [6] and New Zealand [7].

### 1.2. Alcohol Consumption Consequences

Alcohol consumption has unwelcome consequences. Hingson *et al.* [1] analyzed data from 22,224 from several major American studies, including Harvard School of Public Health College Alcohol Survey (CAS) and the National Household Survey on Drug Abuse (NHSDA), and showed that 10.6% of college students reported being hurt or injured because of their drinking, 8.4% having unprotected sex because of their drinking, and 13.3% being assaulted or hit because of other students’ drinking. A large Swedish study has shown that negative consequences are experienced by Swedish students as well: 43% of Swedish university students experienced negative consequences due to their drinking in the past year, with most harm being reported in the physical area (26.3%) and in their financial situation (25.7%) [2].

### 1.3. Alcohol Trajectories

Studies have tried to monitor the drinking habits of students through college and the years following college, to understand how drinking habits develop though those stages in life. This period of life is often dominated by change—starting college and graduating, travel, and eventually having a stable work life, getting married and becoming a parent—changes that bring greater personal responsibility and definition as an adult. This period of change before adulthood, between the ages 18 and 25, has been named “emerging adulthood”, and corresponds well to the period of increased drinking [8,9].

Several studies have shown alcohol consumption patterns that increase until the age of 21, with women having a lower alcohol peak than men, and then slowly decreasing [9–11]. In a Swedish study of university freshmen presenting cross-sectional data, the highest AUDIT (Alcohol Use Disorders Identification Test) score means could be seen within the 24–25 age group for men (peaking at 10.5 ± 5.4 points), and 20–21 years for women (peaking at 7.1 ± 4.3 points; [12]). Donovan *et al.* [13] showed that, of those being problem drinkers in college, 50% of the men and 80% of the women were non-problem drinkers six years later. The findings of Jackson *et al.* [14] support the view that students mature out of their risky drinking habits, with 55% of large-effect drinkers in year one still belonging to that group in year seven.

In recent years, trajectory analyses have been performed to improve the understanding of changes in alcohol drinking across the years. The newer trajectory statistics can combine variable-centred and pattern-centred approaches to form the basis and explanation of data [15]. Several studies have used trajectory analyses within the field of college student drinking [16–19].

Most studies identified stable trajectories of different types of drinking pattern (no-, low-, medium- and high-consumption groups), as well as a group of increasers and a group with temporary high consumption (“fling”). Only Schulenberg *et al.* [17] identified a group with decreasing alcohol trajectories among persons in this age group.

### 1.4. Risk Factors of Drinking Trajectories

It is important not only to identify different drinking trajectories across the college years, but also to identify factors separating the persistent high-risk drinkers from those with non-risky alcohol habits and from those whose at-risk drinking habits decrease after the college years. The most common characteristic of a student belonging to a heavy drinking trajectory group is the male gender [10,14,17,20,21]. Aertgeerts *et al.* [22] found that more students diagnosed with alcohol abuse or alcohol dependence failed their first year in college than other students. Singleton [23] found alcohol consumption to significantly correlate with academic performance in college students, even when controlling for variables such as gender, partying, academic class and parents’ income, as well as SAT (standardized test for college admissions) scores and class rank. Using data from the College Alcohol Study waves, Williams *et al.* [24] found that drinking has a small direct positive effect on GPA (Grade Point Average) scores, but that this direct positive effect is outweighed by a larger negative effect on GPA due to fewer hours spent studying when consuming alcohol. Paschall and Freisthler [25] did not find any relationship between academic performance and measures of heavy alcohol use or alcohol-related problems, nor did Wood *et al.* [26].

Baer *et al.* [3] found Greek house involvement as being a risk factor for heavy drinking, and Schulenberg and Maggs [27] found that students involved in fraternities and sororities were over-represented in chronic, increase and time-limited heavy drinking trajectories and under-represented in the trajectory group that never experienced heavy episodic drinking. Using data from the Monitoring the Future project, McCabe *et al.* [5] examined alcohol use by fraternity and sorority members from the age of 18 to 22. Results showed that those students who were members of fraternities and sororities were more inclined to engage in heavy episodic drinking than non-member students. Sher *et al.* [28] examined the alcohol habits of Greek house members in college and up to three years post-college, and found the relationship between Greek house members and heavy drinking was apparent during the college years, but not thereafter. They also found peer alcohol use norms to partially account for the Greek house members’ heavy drinking during the college years, but no association between heavy drinking and alcohol outcome expectancies, or academic ability. Peer norms is a concept widely used in college student and alcohol studies, where it is hypothesized that the drinking habits of one’s peers—both the actual and the perceived drinking habits—influence the individual’s alcohol consumption. Using data from the same study as Sher *et al.* [28] but with a follow-up period of an additional four years post-college, and using trajectory analyses, Bartholow *et al.* [29] showed that heavy drinking decreased in the post-college years. Furthermore, the drinking trajectory slopes declined more for students heavily involved in the Greek house system. No inclining or stable trajectories could be identified. The majority of the decrease in heavy drinking appeared during the first three years after graduation. Peer norms were associated with heavy drinking, and even eliminated the influence of gender in the trajectory models.

Earlier studies indicate that the social context of the residence halls is associated with the level of alcohol consumption. Oostveen *et al.* [30] showed that two of the factors associated with predicting heavy drinking in young adults were social norms of family and peers as well as socializing. Using the University Residence Environmental Scale, Holle [31] showed that low-drinking fraternity houses scored higher on academic achievement, intellectuality and student influence. A Swedish-originated instrument, Family Climate, measures the social and environmental climate of groups of persons living closely together in family-like environments [32]. This instrument has shown correlations with Moos’ Family Environment Scale [33,34], and has been used in studies and clinical contexts with alcoholics and children of alcoholics (Söderlind and Johnsson, 2004). In unpublished observations of the residence hall student populations on which this paper is based, correlations have been found between the Family Climate and AUDIT. In residence halls where students report higher Distance and higher Expressiveness values (two of the Family Climate scales) compared to other halls, significantly more students also report at-risk AUDIT levels of drinking (OR = 2.4).

Most studies have been carried out in English-speaking countries, with a heavy over-representation of US studies. The Scandinavian countries, including Sweden, have the same dry alcohol culture as the English-speaking world [35], with a high drinking rate on weekends and holidays, but a low drinking rate during the working weeks. Sweden has been shown to have similar drinking patterns to the college student population in the US, with high alcohol consumption amongst freshmen students in both countries. Although the Greek system does not exist in Sweden, it has been shown that Swedish residence hall students have alcohol consumption patterns more similar to American Greek house students than American residence hall students [36]. However, little is known about drinking trajectories of Swedish college students. Johnsson *et al.* [37] followed Swedish university freshmen for four years, with a mean baseline age of 21.3 years. They found that 16% of the students belonged to stable high trajectories, 11% decreasing, 13% increasing, and 60% in stable low trajectories, corresponding well to American studies. No previous studies on alcohol trajectories in the Swedish residence hall population have been performed. A difference between English-speaking countries and Sweden in this context is that the age of college students in Sweden is generally higher than in the English-speaking countries. Since the question of emerging adulthood and maturing out of risky alcohol habits is closely related to age as well as to social contexts, it is important not to directly extrapolate data from the English-speaking countries to Sweden without further Swedish research in this field.

### 1.5. Aim and Hypothesis

This study attempts to follow the drinking habits of students in Swedish university residence halls over three years, as part of an alcohol intervention study. The intervention study compares two interventions (a Brief Skills Training Programme, BSTP, intervention and a Twelve Step Influenced, TSI, intervention) to a control group, and is further explained in section 3.5. Covariates were added to the drinking trajectories to study the effect of age, gender, residence hall social environment, academic success and type of alcohol intervention on the drinking trajectories of those students. From earlier studies, we expected to find gender, age, residence hall environment and alcohol intervention at baseline to be predictors of drinking habits, but no relationship with academic success. We also expected the analysis to include increasing as well as decreasing and stable trajectory patterns.

## 2. Results and Discussion

### 2.1. Baseline Results

At baseline, the mean (± sd) of AUDIT was 10.8 ± 5.0 for men, and 8.0 ± 4.4 for women. SIP scores were 3.8 ± 3.0 for men and 2.9 ± 2.6 for women. eBAC estimates were 1.10 ± 0.66 for men and 1.06 ± 0.74 for women.

### 2.2. Identification of Trajectories

Trajectory groups were detected using Bayesian Information Criteria (BIC) values as determinants of the number of groups used (Table 1).

The best-fit models contained five groups for AUDIT, four groups for SIP and three groups for eBAC. The best-fit model for AUDIT is linear for groups 1, 3 and 5, and quadratic for groups 2 and 4. As for SIP and eBAC, all group models are linear.

#### AUDIT (Alcohol Use Disorder Identification Test)

**Figure 1.**
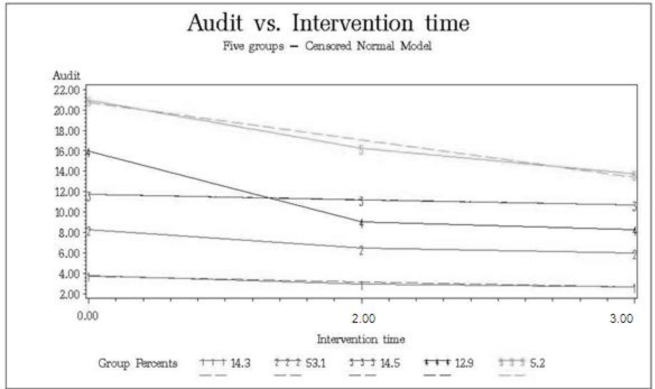
AUDIT trajectories including year 0, 2 and 3 (no data is available for year 1, see section 3.4). Trajectory groups: stable low (1; 14.3%), medium decreasing (2; 53.1%), stable high (3; 14.5%), high decreasing (4; 12.9%) and very high decreasing (5; 5.2%). Solid lines represent actual values, dotted lines represent fitted values.

Five different trajectory groups were identified, as shown in Figure 1. While all groups decreased their scores across the years, the groups called stable only show minor decreases. The identified trajectory groups were: stable low (14.3%, with a mean decrease of 1.2 points across the years), medium decreasing (53.1%, mean decrease 2.3), stable high (14.5%, mean decrease 1.0 points), high decreasing (12.9%, mean decrease 7.7) and very high decreasing (5.2%, mean decrease 7.3).

#### SIP (Short Index of Problems)

**Figure 2.**
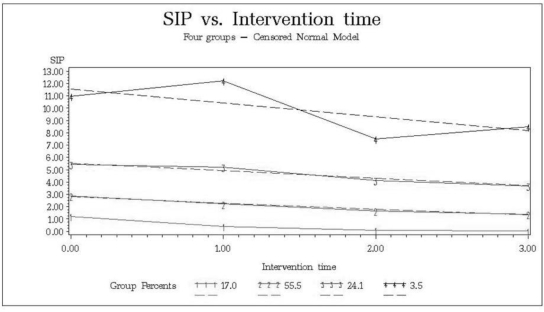
SIP trajectories. Groups: Stable low (1; 17.0%), stable medium (2; 55.5%), stable high (3; 24.1%), very high decreasing (4; 3.5%). Solid lines represent actual values, dotted lines represent fitted values.

All four groups best fitting the trajectory model and SIP scores across the years decreased their SIP scores. The four defined trajectory groups included stable low (group 1 in Figure 2; 17.0%, with a mean decrease of 3.8 points across the years), stable medium (55.5%, mean decrease 1.3), stable high (24.1%, mean decrease 1.2) and very high decreasing (3.5%, mean decrease 2.3). The high decreasing group showed increases in SIP scores for years 1 and 3, but showed an overall decrease.

#### eBAC (estimated Blood Alcohol Concentration)

**Figure 3.**
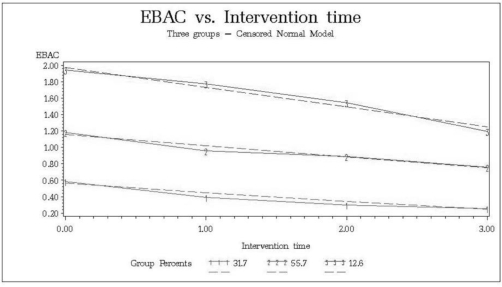
eBAC trajectories. Groups: low decreasing (1; 31.7%), medium decreasing (2; 55.7%) and high decreasing (3; 12.6%). Solid lines represent actual values, dotted lines represent fitted values.

Three trajectory groups were defined for eBAC across time, and all of those groups decreased their eBAC levels with time (Figure 3): low decreasing (group 1 in Figure 3; 31.7%, with a mean decrease of 0.3% across the years), medium decreasing (55.7%, mean decrease 0.3) and high decreasing (12.6%, mean decrease 0.5).

### 2.3. Covariate Analyses

After analysis of the separate trajectories for the three different drinking instruments, univariate analyses were performed on each of them. The independent variables added included age, gender, academic success, the four Family Climate subscales closeness, distance, expressiveness and chaos, and intervention group.

#### AUDIT

Age and gender were significant for group membership in most groups (see Table 2). No women were found in the very high decreasing AUDIT trajectory. The lowest trajectory group of AUDIT had the highest mean age, and included more women. Students with low closeness were more likely to belong to the very high decreasing trajectory group. Students with high expressiveness were more likely to belong to the lowest trajectory group. Distance and chaos were not significant, nor were the other covariates, including academic success and intervention groups.

#### SIP

Both age and gender were significant for most trajectory group membership (see Table 2), with lower trajectory groups more likely to include older students and more women. Students reporting high expressiveness were more likely to belong to the lowest trajectory group. Academic success was significant for belonging to the high stable group compared to the low stable group.

#### eBAC

Lower age significantly predicted membership of the medium decreasing group, see Table 2. Gender did not have a significant influence. Higher levels of chaos and higher levels of academic success predicted membership in the medium decreasing group.

### 2.4. Adjustments

Since age and gender were significant in all three drinking instruments and in almost all trajectories, the significant covariance analyses described above were re-run, adjusted for age and gender. All significant differences except one—low expressiveness predicting membership in the SIP stable medium group—then became non-significant.

### 2.5. Discussion

As previous studies have shown, drinking habits change over the years and are suitable for trajectory analyses.

In most trajectories, male gender and lower age predicted membership in the higher drinking group trajectories. Higher alcohol consumption is common in younger males and has been reported in most previous studies [10,14,16–21].

An interesting observation is that the relationship between age and membership of a higher trajectory group only reaches significant levels in the low- and mid-level trajectory groups and not in the highest ones. This is also true for gender and trajectory group membership when it comes to eBAC. Persons with genetic risk factors for alcoholism are shown to have a lower level of response to alcohol [38–41]. A low level of response to alcohol at age 20 predicts the later development of alcohol abuse or alcohol dependence [38]. Perhaps the highest eBAC trajectory groups include persons with low levels of response, having found they have to drink larger amounts of alcohol in order to have the same effects as other persons. This would then reflect a persistent pathological relationship to alcohol in the highest trajectory groups.

In this study, no groups were found with increasing alcohol habits measured with AUDIT, SIP and eBAC. This finding is consistent with the findings of Bartholow *et al.* [29], studying students in the Greek house system in the US. The reason for this might be multifactorial.

The mean age of the students included in the study was 23.2 years at baseline, which is two years older than the mean freshman age that year. In a similar study of freshmen engineering students at the same university, increasers were found [37]. It has been shown that students have higher alcohol consumption during their first year in college [2], and from the mean age it can be hypothesised that the students included in this study were past the freshman year. Thus, it is possible that no increasing trajectory could be identified because the year of highest alcohol consumption had already passed and most students were at, or had already passed, the peak of their consumption curve at the beginning of the study. In the engineering freshmen study [37], the highest trajectory group was found at around AUDIT score 20, and in our university residence hall study the high decreasing group in AUDIT started at a score of 20.7, further supporting this hypothesis. Unfortunately, no questions were asked about the year of study of the student. Other trajectory studies have found decreasing groups, as well as fling trajectory groups, where an increase in the measured variable is followed by a decrease [41,42]. This is consistent with our findings, especially if the current study, as discussed above, caught the students at the top of a hypothetical fling pattern in the baseline measurements.

Another possibility is that the students were affected by the study and the mailed minimal feedback after each questionnaire, and that their alcohol drinking habits decreased as a consequence of this. Regardless of the intervention randomization, all students completing the questionnaires each year received a mailed minimal personalized feedback. Research has shown that mailed personalized feedback influences the students’ alcohol habits, especially when normative feedback was included [43]. Although the design of the personalized feedback in this study was indeed minimal, no assessment-only group was included, and it cannot be excluded that the mailed personalized feedback had an effect on the trajectory pattern.

In this study, one of the inclusion criteria was living in a residence hall at the initiation of the study. It is probable that at least some of the students moved out during the course of this three-year study. It is also shown, as discussed above, that students living in residence halls have higher alcohol consumption than other students. The finding of only stable and decreasing trajectories could thus be partly due to students moving out of the residence halls, changing their alcohol habits to fit their new living environment.

Academic success was not found to have any impact on the trajectory groups. As mentioned in the introduction, effects on academic success have been mixed in previous studies, and there is no uniform conclusion on academic results and alcohol use. More research is needed into this particular field before any conclusions can be drawn about students’ alcohol consumption and the correlation with academic performance.

It can also be seen that the social context (as measured by Family Climate) of the residence halls has some impact upon alcohol trajectories, but that this impact is eliminated by age and gender. No known trajectory studies have measured the social climate of the living arrangement previously, but peer influence is a related factor, shown to influence students’ alcohol habits. However, peer influence as such is not measured in this study.

### 2.6. Strengths and Weaknesses

It has previously been shown that Swedish residence halls have a high proportion of at-risk drinkers, not unlike American students engaged in Greek houses. It is thus of great interest to follow the drinking trajectories of the Swedish residence hall students, which has not been done previously. A semi-parametric trajectory analyses is used, making optimal use of the data available. Sufficient power to include covariates in the trajectory analyses is another strength that adds to the explanatory value of the article.

Our social context questionnaire, measuring the perceived social climate in the individual residence halls, does not directly measure alcohol-related climates and peer use of alcohol. This is one of the weaknesses of our study. Other weaknesses are the lack of AUDIT data from year one, making this trajectory analysis more uncertain than the other two, and the exclusive reliance on self-reported data. Furthermore, since all students completing the questionnaires received mailed normative feedback, including the students randomized to a control group, it might be argued that the control group is not, in fact, a control group. This might conceal differences between the trajectory groups with respect to interventions.

## 3. Experimental Section

### 3.1. Participants and Enrolment

All residence halls within the University of Lund (n = 271) were orally invited to participate in the study, through a student representative. The residence halls accepting this invitation (n = 252) were given in-depth information about the study, and those students willing to participate signed a consent form and completed a baseline questionnaire. Ninety-eight halls of residence, where over 50% of the inhabitants accepted inclusion, were cluster randomized (with the residence hall unit as the basis of randomization) to three different intervention groups. Follow-up questionnaires were mailed once a year for three years, and non-responders were reminded either by post or by telephone calls. A more detailed description of the study has been published elsewhere [6].

The study was approved by the ethics committee at Lund University.

### 3.2. Follow-up Rates

A total of 556 students were cluster randomized at baseline. At first-year follow-up, 405 (72.8%) students answered the questionnaire, at second-year follow-up, 371 (66.7%) students completed the questionnaire, and at third year follow-up, 363 (65.3%) students completed the questionnaire. 304 students (54.7%) answered all four questionnaires.

### 3.3 Initial Data

At baseline, the mean age was 23.2 years and 64.2% of the participants were male. No differences were seen between the three groups in age or gender distribution. In the year the study was started, the mean freshman age at the University of Lund was 21.3 years, and 45% of the freshmen were male [44].

### 3.4. Measures

*AUDIT*—Alcohol Use Disorders Identification Test, was used at baseline and in the second and third follow-up questionnaires (omitted at first follow-up due to human error). It was developed by the World Health Organization in the 1980s [45], and has since been used worldwide in both clinical and research settings, to measure alcohol consumption, harm and dependence symptoms. AUDIT consists of ten questions, each scored from 0 to 4 points. The maximum score is thus 40 points. NIAAA [46] have recommended cut-off scores of eight or above for men and four or above for women, indicating at-risk drinking. The Swedish version [47] of the instrument was used, which has been validated and found to have a Cronbach’s alpha of 0.81 [48]. Cronbach’s alpha in this study was 0.84. In the Swedish version, a standard drink is defined as containing 12 grams of alcohol. AUDIT is a valuable screening tool for alcohol use problems, and is increasingly used in studies within the student population. Inclusion of an AUDIT trajectory thus increases the understanding of the drinking trajectories, and allows direct comparison to other studies.

*SIP*—Short Index of Problems is a shorter version of DrInC (The Drinker Inventory of Consequences; [49] and has been used in previous studies in college student alcohol prevention research in Sweden [50]. It has 15 questions and a maximum score of 45, and measures a wide variety of consequence areas: physical, intrapersonal, social responsibility, interpersonal and impulse control. It has been translated to Swedish by the Clinical Alcohol Research group at Lund University. Miller *et al.* [49] obtained an internal consistency of 0.81 and, in our study, Cronbach’s alpha was 0.91. This questionnaire was used in the baseline questionnaire and all follow-ups.

*eBAC*—estimated Blood Alcohol Concentration, is a retrospective self-report measure of the estimated blood alcohol concentration, calculated from the given gender, body weight, hour of drinking and number of standard drinks consumed on the last pleasant drinking occasion, using the formula given by National Highway Traffic Safety Administration [51], which is used in previous similar studies [12,50]. The wording “pleasant” drinking occasion is used in previous Swedish student alcohol studies [12,50], and is chosen to represent an optimal drinking occasion rather than a peak drinking occasion. eBAC is reported in grams per litre, as common in Sweden. This differs from the American units (g/dl) by a factor of ten. This questionnaire was used in the baseline questionnaire and all follow-ups.

*Family Climate*—Constructed by Hansson [33], this instrument measures the perceived social climate in family-like settings in four different dimensions: closeness, distance, expressiveness and chaos. A list of words is given, and the words perceived appropriate for the measured environment are to be underlined. A ratio is calculated, where numbers above one indicate that more words have been underlined in that particular dimension than on the scale as a whole. This instrument is widely used in clinical practice in Sweden, is thoroughly validated and has been used in several research studies, including substance abuse research [32]. Cronbach’s alpha has been shown to be 0.98 for closeness, 0.91 for distance, 0.71 for expressiveness and 0.92 for chaos [33]. This questionnaire was only used at baseline.

*Academic success* — in this self-report questionnaire, students report the credits achieved during the past year, and the maximum possible number of credits that could have been achieved. From this, academic success could be derived, defined as having achieved 75% or more of the possible credits (using the same definition as the Swedish Student Loan Foundation) across the four measuring times. This questionnaire was used in all four years of the study.

### 3.5. Alcohol Interventions

For a more complete description of the interventions, see Stahlbrandt *et al.* [6]. All students completing the questionnaires, including those excluded before randomization at baseline, received a mailed feedback containing their scores on AUDIT, SIP and eBAC, in relation to the mean score of the whole group. The students were randomized to a BSTP (Brief Skills Training Programme) intervention, a TSI (Twelve Step Influenced) intervention, or a control group. The BSTP was based on the BASICS [52] manual and has been used in previous studies at Clinical Alcohol Research [50,53]. The second intervention, TSI, consisted of therapists from Nämndemansgården, a well-known Swedish 12-step institution, giving a lecture on alcohol and alcoholism, and bringing a person with former alcohol problems helped by the twelve step program, to give a presentation. Intervention group belonging was added as a co-variate in the analyses.

### 3.6. Statistical Methods

Trajectories were identified using the semiparametric group-based model (SGM) described by Nagin [54]. The analysis assumes the population studied consists of a mixture of heterogeneous groups defined by different developmental trajectories, and fits semiparametric mixtures of several distributions including censored normal, to longitudinal data. It is a particularly useful model for repeated measurements, since it only needs two trajectory values to determine parameter estimates, which means a minimal data loss. In the SAS/TOOLKIT computer program, data is analyzed and sorted into different trajectory groups. Each individual is then assigned to a group, depending on the individual’s fit in the different groups. BIC (Bayesian Information Criteria) values are analyzed in order to determine the number of trajectory groups best fitting the data, where smaller absolute values indicate a better fit [55,56]. SGM have previously been used for alcohol trajectories [15,19–21,37,57,58].

Trajectory groups were created from three different instruments: AUDIT, SIP and eBAC.

Independent variables were individually added to the analysis as covariates. Those included age, gender, academic success, the four Family Climate subscales closeness, distance, expressiveness and chaos, and intervention group. The age variable was dichotomized to above and below mean age (*i.e.*, 24 years and above, or 23 years and younger), since continuous co-variates could not be used in semi-parametric trajectory analyses. The variables that were significant (at the 0.05 level) in this analysis were put through a multivariate analysis, including gender and (dichotomized) age as covariates. The different groups were compared to the base group, the lowest one, for each of the three instruments AUDIT, SIP and eBAC. The trajectory program handled missing data across the years.

## 4. Conclusion

In an analysis of alcohol trajectories in this high alcohol-consuming group, no trajectories of increasing alcohol habits could be found. This might be due to normal development, or due to alcohol interventions given in the first year of the study.

## Figures and Tables

**Table 1.** BIC (Bayesian Information Criteria) values for different numbers of trajectory groups.

*No. of groups*	*AUDIT*	*SIP*	*eBAC*
2	−3590.23	−3680.19	−1677.66
3	−3546.44	−3611.24	**−1662.36**
4	−3518.86	**−3599.35**	No acceptable models
5	**−3511.08**	−3607.47	No acceptable models
6	No acceptable models	Not tested	Not tested

**Table 2.** Age and gender as covariates in different trajectories, predicting group membership. All groups compared to the lowest group. Multinomial logit coefficient estimate (p-value).

		*Age*	*Gender*
**AUDIT**	Medium decreasing	−**0.71 (0.04)**	**1.15 (0.00)**
	Stable high	−**2.14 (0.00)**	**2.87 (0.00)**
	High decreasing	−0.26 (0.52)	**1.70 (0.00)**
	Very high decreasing	−0.42 (0.40)	- (no women)
**SIP**	Stable low	**−0.80 (0.02)**	0.62 (0.08)
	Stable medium	**−1.45 (0.00)**	**1.26 (0.00)**
	High decreasing	−0.60 (0.28)	**1.80 (0.02)**
**eBAC**	Medium decreasing	**−0.79 (0.00)**	0.01 (0.97)
	High decreasing	−0.75 (0.57)	0.07 (0.86)
